# The association between weather warnings and hip fractures in the Republic of Ireland

**DOI:** 10.1007/s11657-023-01243-9

**Published:** 2023-04-21

**Authors:** Ciarán Stanley, David Lennon, Frank Moriarty, Gerard A. Sheridan, Matthew Nagle, Alexandra Foley, Conor Moran, Michael Donnelly

**Affiliations:** 1 Department of Trauma and Orthopaedics, Beaumont Hospital, Dublin 9, Ireland; 2 School of Pharmacy and Biomolecular Sciences, Royal College of Surgeons in Ireland, Dublin, Ireland; 3 Department of Trauma and Orthopaedics, University Hospital Limerick, Limerick, Ireland

**Keywords:** Hip fractures, Trauma, Trauma systems, Weather warnings

## Abstract

***Summary*:**

This study examined the relationship between hip fractures and weather warnings with the hypothesis higher rates of fractures occur during warnings. National hip fracture database and weather warning records were utilised. Higher rates of hip fractures were found with severe warnings. This has implications for planning in future severe warnings.

**Background:**

Hip fractures represent a significant burden on the Irish Health system with 3666 in 2020. The Irish National Meteorological Service operates a colour coded warning system. Yellow being least severe, while orange represents weather with capacity to impact individuals in affected areas and red represents advice to protect themselves and property. Previous studies investigated the seasonality of hip fractures, which remains but none have investigated the relationship between weather warnings and rates of hip fractures. The hypothesis was that increasing weather warnings would be associated with increased hip fractures. The aim was to investigate the relationship between weather warnings and hip fractures in the Republic of Ireland.

**Methods:**

Comparison of national weather warning archives from 2013 to 2019 to Fracture Database records. Reviews assessed whether fractures occurred on days a weather warning was in place in the individual’s local county. A statistical analysis of warning features and their relationship to hip fractures was then performed. Fractures and weather warnings were stratified by county with both a panel and case crossover analysis performed.

**Results:**

There was a tendency towards increased incidence of hip fractures with weather warnings in adjusted analysis (IRR 1.02; 95%CI 0.99–1.06; *p*-value 0.123). Orange warnings were associated with a statistically higher incidence of fractures (IRR 1.06; 1.01–1.12; *p*-value 0.026). In both panel and case crossover analysis, both orange and yellow warnings were associated with fractures. Red warnings were associated with a lower incidence of fracture on day of warning (adjusted incidence rate ratio 0.92; 0.70–1.22; *p*-value 0.569) but a higher incidence on the following day (adjusted incidence rate ratio 1.14; 0.88–1.46; *p*-value 0.313).

**Conclusion:**

An increased incidence of hip fractures appears to occur during weather warnings. Consideration should be given when preparing for periods of extreme weather, ensuring sufficiently resources.

**Supplementary Information:**

The online version contains supplementary material available at 10.1007/s11657-023-01243-9.

## Introduction

 Osteoporotic hip fractures are a common injury presenting to orthopaedic units internationally and are associated with significant morbidity, mortality, cost and reduced quality of life [1]. Hip fractures in Ireland represent a significant burden on the Irish Health system with a total of 3666 hip fractures in 2020 [2] accounting for 2% of the Irish healthcare budget in 2019 [3].The average length of stay for these patients was 17.1 days in 2019 [4]. The estimated cost of a single Hip fracture over 12 months post injury is $43,669 [5] which is significantly more than the cost for acute coronary syndrome ($32,345) and ischaemic stroke ($34,772) over a similar period [6, 7]. In Ireland the cost of care of patients with hip fractures in 2019 is estimated to have been 45 million euro, which accounts for roughly 0.2% of the national healthcare spending. The Irish Hip Fracture Database (IHFD) has been collecting data on all geriatric hip fractures occurring in Ireland since 2013. It was set up through the National Office of Clinical Audit (NOCA) and publishes reports annually.

Weather events are becoming increasingly frequent as a result of global warming and climate change, and these present a significant health risk [8–11]. Differing injury patterns from weather disasters have been noted in many international settings following tsunamis, earthquakes and many other large scale natural events [12–16]. It is clear that weather can play a significant part in trauma presenting to hospitals; however, only a limited number of studies to date have assessed the impact of local weather warnings, events and variables on trauma presentations to Emergency Departments [17–20].

The Irish meteorological service operate a colour coded weather warning system to inform citizens of the level of risk of incoming weather fronts. This colour coded system was introduced in 2012, with red being the most severe, followed by orange and then yellow [21]. Advice for incoming red warnings is to protect oneself and your property due to the severity of the incoming weather, while orange warnings represent advice to be prepared as incoming weather has the capacity to significantly impact people’s lives. More detailed information on specific weather warnings and the parameters are available on the Meteorological Society website, however while thresholds exist for each weather warning the decision to implement a warning is primarily made off the perceived impact and risk to population of incoming weather. Government data archives provide a record of all-weather warnings, issued on a county-by-county basis from the 25th of April 2012 to the 31st of December 2019[22].

Previous studies have examined the relationship between hip fractures and air temperature [23], snow levels [24] and seasonality [25, 26]. In a systematic review published on the topic, low temperature, snow, ice and low sun exposure are all associated with higher rate of hip fractures [27]. None of these publications have focused on weather warnings or utilised national hip fracture databases and weather warning systems to investigate their relationship. The hypothesis of this research was that increasing weather warnings would be associated with increased rates of hip fractures.

## Aim

The aim of this study is to assess the relationship between weather warnings and hip fracture rates in the Republic of Ireland.

## Methods

Ethics for this project was sought and approved by the ethics committee in our local hospital (REC reference no. 20/65) and a data request was submitted to the National Office of Clinical Audit (NOCA). Following NOCA approval, access was granted to the IHFD which included data between the years 2013 and 2019. Details included within the database included fracture pattern, date of presentation to ED and the county in which the patient resided.

A retrospective review of 7 years of data from the IHFD base and the Irish National Meteorological service warnings was performed, from January 2013 to December 2019. Hip fractures were divided based on patient’s county of residence. Days affected by weather warnings were the dates specified in the notice issued by the National Meteorological Service, as well as the following day, given there may be a delay between a hip fracture occurring and presenting for treatment. This delay may be more likely during an extreme weather event.

Incidence rates were calculated using the population of people aged 50 years and over from the Census 2016 for each county [28], and days affected by weather warnings in each county. A calculation of numbers of person-days affected by weather warnings was made. The number of hip fracture admission on those days were used to calculate incidence rates of hip fracture admissions per million person days not affected by weather warnings and affected by weather warnings. Using rate per million non-warning days as the reference group incidence rate differences and incidence rate ratios were calculated, with 95% confidence intervals, for warning days, red warning days, orange warning days, and yellow warning days. All *p*-values were calculated using mid-p adjustment and statistical significance was assumed at a value of less than 0.05.

Using panel data, an assessment of whether hip fractures at the county level per day were associated with weather warnings affecting that day was performed. This ecological analysis approach accounts for the temporal structure of the data. A fixed effects Poisson panel regression model was used, including variables for severity of warnings affecting that day and county. As the outcome variable was a count of hip fractures per day, and there was no evidence of over-dispersion in the distribution of the outcome variable, a Poisson model was selected over a negative binomial model. Variables were included to adjust for the warning element, as the type of weather event could affect the warning severity as well as fracture incidence. County population aged 50 years and over was the exposure variable, and the analysis was adjusted for season and year. Parameters were estimated as adjusted incidence rate ratios with 95% confidence intervals.

A time-stratified case crossover design was also utilised. This is a case-only study design, making use of data on only individuals with a fracture admission, where participants serve as their own control. In this analysis, a comparison of any weather warnings affecting the case day to those affecting the control days was performed. The case day was defined as the date of hip fracture admission for each individual. Four control days were used for each case, being 7 and 14 days before and after the case day (considering weather warning affecting the county of residence). Fractures occurring in the first or last 14 days of the study period (January 2013 to December 2019) were excluded. This ensures comparisons were based on the same day of week and account for seasonality and prevailing weather conditions either side of the case day. This can control for both county and individual level factors that may confound the relationship between weather conditions and hip fracture admissions. For this analysis, conditional logistic regression was used, including variables for severity of warnings, and adjusting for the warning elements, matching case and control days for each individual. Parameters were estimated as adjusted odds ratios with 95% confidence intervals.

Sensitivity analyses were conducted for both the panel analysis and case crossover analysis, examining whether warning parameters where a warning was assumed to affect only the exact dates it was in place, and second, assuming a lagged effect of weather warnings (a warning applying to a Monday and Tuesday was considered to affect the Tuesday and Wednesday).

Last, an examination was carried out of the relationship between warning element and hip fractures, using a similar approach as used for warning severity (named calculating unadjusted incidence rates and IRR, panel analysis adjusting for season and year, and case crossover analysis). All analyses were conducted with Stata version 17.

## Results

**Fig. 1:**
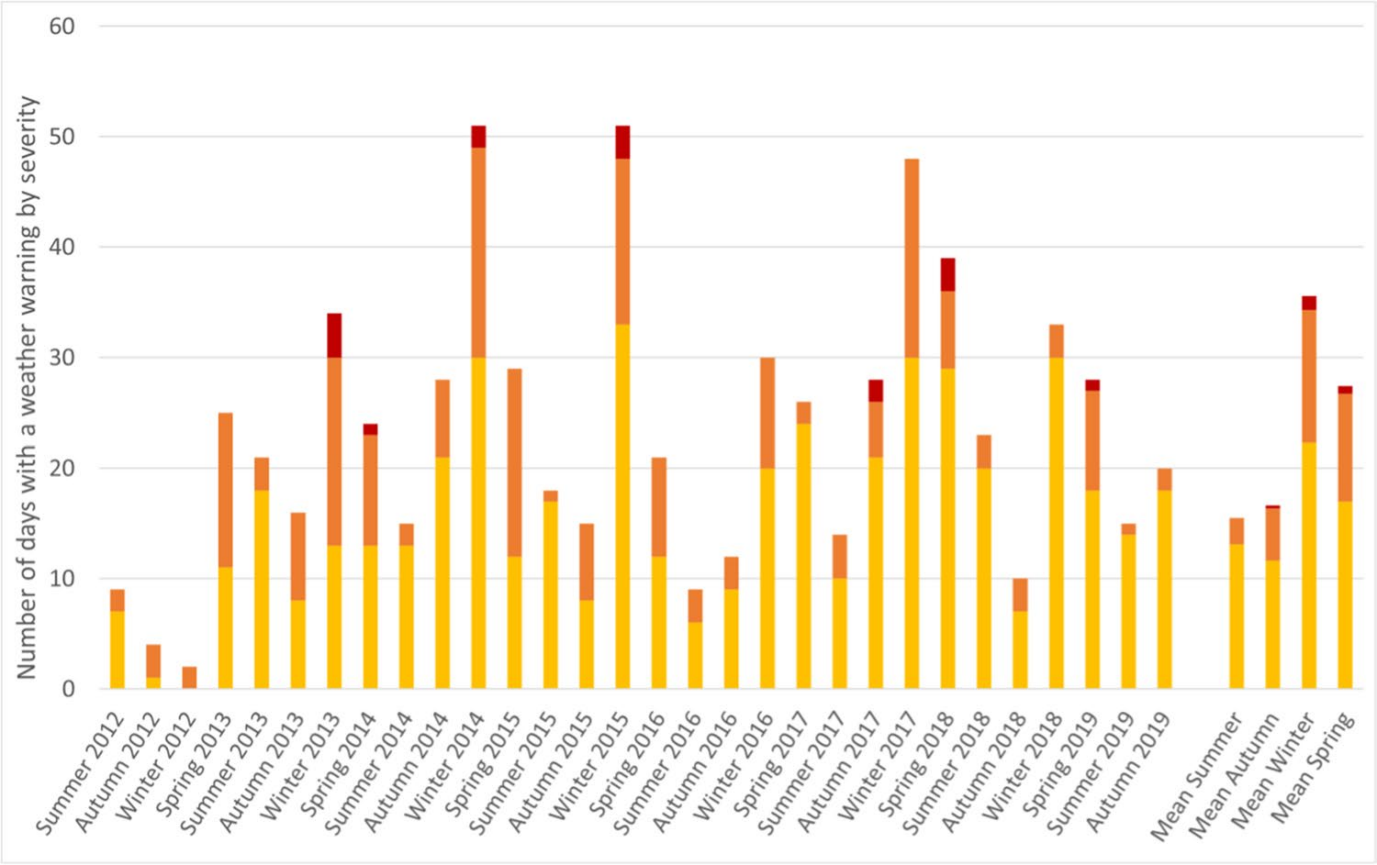
Number of days per season with a weather warning in place by severity and mean weather warnings per season

**Fig. 2:**
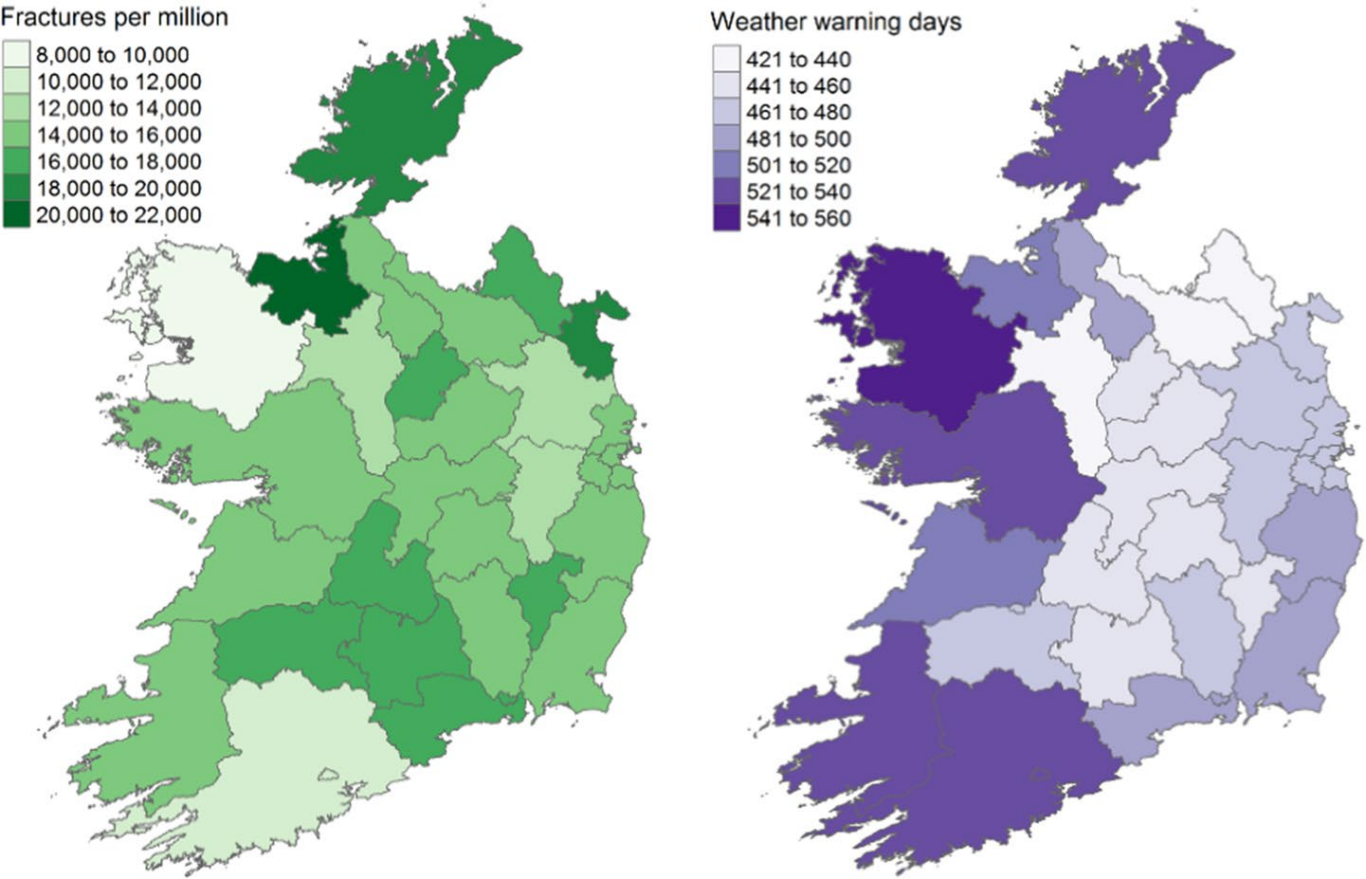
Comparison of counties by rate of hip fracture admissions per million population aged 60 years and over (left) and the number weather warning days (right)

The number of days per season where a weather warning was in place in Ireland during the study period is shown in Fig. 1, ranging from 2 days (Winter 2012) to 51 days (Winter 2014 and 2015). The mean number of warning days was highest during winter, followed by spring, and yellow warning were most common. Over the full study period, rate of hip fracture admissions by county ranged from 8927 to 20,894 per million population aged 50 years and over, and total number of days with a weather warning by county ranged from 436 to 545 (Fig. 2). The crude incidence rate of hip fracture admissions was 5.89 per million person-days not affected by a weather warning (Table 1). This compares to 5.99 per million person-days affected by a weather warning, however this difference was not statistically significant (unadjusted incidence rate ratio 1.02, 95% CI 0.99 to 1.05). Comparing each level of warning, the incidence rate were similar for days affected by a yellow or red warning compared to no warning, however the incidence rate was significantly higher for orange warning (IRR 1.06, 95% CI 1.01 to 1.12; *p*-value 0.032).

**Table 1:** Summary of hip fracture events and unadjusted incidence rates for days affected by weather warnings by severity

	Events	Exposure (million person days)	Incidence rate	Incidence rate difference (95% CI)	Incidence rate ratio (95% CI)	*p* value
No warning	15,850	2688.8	5.89	–	–	–
Any warning	5868	978.9	5.99	0.13 (− 0.05 to 0.30)	1.02 (0.99 to 1.05)	0.169
Warning severity						
Red	106	18.1	5.84	− 0.05 (− 1.17 to 1.07)	0.99 (0.81 to 1.20)	0.941
Orange	1498	239.7	6.25	0.35 (0.02 to 0.68)	1.06 (1.01 to 1.12)	0.032
Yellow	4264	716.8	5.95	0.05 (− 0.15 to 0.25)	1.01 (0.98 to 1.04)	0.600

Considering panel data analysis at the county level, adjusting for type of warning, season, and study year, all levels of warning severity were associated with a higher incidence rate of hip fracture admissions compared to no warning, although this only reached statistical significance for yellow (adjusted IRR 1.10, 1.02 to 1.19; *p*-value 0.02) and orange warnings (1.24, 1.12 to 1.37; *p*-value < 0.001).

Similarly using a case crossover analysis, again adjusting for warning type, there was higher odds of an individual having a hip fracture admission on days affected by a yellow (adjusted OR 1.13; 1.03 to 1.23; *p*-value 0.007) or orange warning (1.24; 1.11 to 1.39; *p*-value < 0.001) (Table 2). The odds for red warnings were similar to no warning being present. In sensitivity analysis examining the assumption of warnings affecting the period of the warning and the day after, findings were similar for both orange and yellow warnings (Supplementary table 1). These also indicated that the lack of association of red warnings may be driven by a lower incidence on the same days where warnings are in place and a higher incidence on the day immediately following warnings.

**Table 2:** Panel analysis using Poisson regression and case crossover analysis using conditional logistic regression examining the relationship between warning severity and fracture incidence

	Panel analysis		Case crossover analysis	
	Adjusted incidence rate ratio*	*P* value	Adjusted odds ratio**	*P* value
Warning severity				
Red	1.09 (0.88 to 1.35)	0.452	1.01 (0.80 to 1.28)	0.908
Orange	1.24 (1.12 to 1.37)	< 0.001	1.24 (1.11 to 1.39)	< 0.001
Yellow	1.10 (1.02 to 1.19)	0.02	1.13 (1.03 to 1.23)	0.007

^*^Poisson regression with county population aged 50 years and over as exposure, adjusted for season, year, and warning element

^**^Conditional logistic regression matching case and control days for each individual, adjusted for warning element

The most common warning element was wind (416.2 million person days with a wind warning in place) followed by rainfall (345.1 million person days) and snow-ice (186.2 million person days) (Supplementary Table 2). The highest fracture incidence rate was seen for snow-ice warnings (6.48 fractures per million person days), which was significantly higher compared to days with no warnings (unadjusted IRR 1.10; 1.04 to 1.17; p-value 0.002) (Supplementary Table 2). The lowest incidence rate related to high temperature warnings, significantly lower than no warnings days (unadjusted IRR 0.84; 0.73 to 0.97; p-value 0.016). However in panel analysis adjusting for season and year, as well as in case crossover analysis (Table 3), no association between warning element and fractures remained statistically significant.

**Table 3:** Comparison of weather warnings element panel analysis and case crossover

	Panel analysis		Case crossover analysis	
	Adjusted incidence rate ratio*	*P* value	Adjusted odds ratio**	*P* value
Warning element				
Wind	1.01 (0.96 to 1.06)	0.692	0.99 (0.94 to 1.05)	0.821
Rainfall	0.97 (0.92 to 1.01)	0.147	0.96 (0.91 to 1.01)	0.13
Snow-Ice	1.01 (0.95 to 1.08)	0.681	1.00 (0.93 to 1.07)	0.958
Low temperature	1.01 (0.93 to 1.09)	0.835	0.99 (0.91 to 1.09)	0.886
Fog	1.04 (0.95 to 1.14)	0.409	1.03 (0.93 to 1.14)	0.623
Thunder	1.11 (0.97 to 1.25)	0.12	1.13 (0.97 to 1.30)	0.109
High temperature	0.95 (0.82 to 1.09)	0.454	0.97 (0.82 to 1.14)	0.675
Flooding	1.22 (0.76 to 1.94)	0.414	1.14 (0.67 to 1.92)	0.635
Wind	1.01 (0.96 to 1.06)	0.692	0.99 (0.94 to 1.05)	0.821
Rainfall	0.97 (0.92 to 1.01)	0.147	0.96 (0.91 to 1.01)	0.13

^*^Poisson regression with county population aged 50 years and over as exposure, adjusted for season, and year

^**^Conditional logistic regression matching case and control days for each individual

## Discussion

Based on 7 years of national weather warning and hip fracture data, unadjusted analysis showed a tendency towards an association between the presence of weather warnings and a higher incidence of hip fractures with an overall incidence rate ratio of 1.02 (0.99–1.05; *p*-value 0.17) but not reaching statistical significance. A potential reason for this is the weighting of yellow weather warnings, which represent less severe weather and account for over 70% of weather warnings. However, using two different analytical approaches with appropriate adjustment for other factors, we found evidence that orange and yellow warnings were associated with higher fracture rates.

Previous studies have identified higher rates of hip fractures in urban dwellers [29, 30]; however, the incidence is not higher in the counties with Ireland’s four largest cities (Dublin, Cork, Limerick and Galway) when compared to surrounding areas (Fig. 2). Publications showing higher urban rates had hypothesised it may be due to increase number of nursing homes in urban areas but this is also the case in Ireland with over a third of our nursing home beds in the region surrounding Dublin [31]. This difference may be attributable to the increased average age of individuals living in rural Ireland versus urban with an average difference of 2.6 years in 2016 [31]. Our analysis accounted for any county-level effects.

Another potential reason being the majority of falls resulting in hip fractures occurs in the home and this may not display an association with weather [32]. The increased rate on days with weather warnings could be due to advice to decrease travel on days with weather warnings and vulnerable individuals may receive less support than normal as a result, regardless of whether predicted weather occurred. With more severe red warnings advising to strictly remain indoors this may result in carers not travelling to care for vulnerable individuals, more so than would be the case in less severe yellow warnings. There is a higher level of hip fractures associated with orange weather warnings (incidence rate 1.06; 1.01–1.12; *p*-value 0.03), representing more severe weather but not with red warnings (incidence rate 0.99; 0.81–1.20; *p*-value 0.94), the most severe. This may be accounted for in part by the limited number of red warnings, with red warnings only in place for 17 days over the study period, and a larger sample size could display a more significant difference. Of note there was a higher incidence of hip fractures presenting to hospitals the day following a red warning (IRR 1.14; 0.88 to 1.46; *p*-value 0.313) (Supplementary Table 1), which could be accounted for by carers not travelling on the day of warning causing late identification of these injuries in those requiring care. Overall higher rates of hip fractures occurred with red and orange warnings compared to days of no warning and days of yellow. The higher incidence of hip fractures in orange and red warnings suggest that higher weather warnings, irrespective of severe weather occurrence, may increase rates of hip fractures.

In examining the various warning types there was a weighting of these element warnings towards yellow warnings. Although some warning elements had an association with fracture incidence in unadjusted analysis (i.e. snow-ice and high temperature warnings) this was likely confounded by a time of year effect. In analysis accounting for season and year, no evidence of a significant association with hip fracture incidence was found. Multiple prior studies on the relationship between weather and fractures have shown higher rates of fractures in elderly [18] and in hip fractures [25] during winter months when compared to Summer and Spring, which may be up to 15% higher for hip fractures [17].

Previous publications have had varying results when investigating the relationship between weather and hip fractures. Some have found increasing rates of hip fractures in association with lower temperatures [23–25], seasonality [17, 18, 25, 26] and daily duration of wind [33]. While this study did not examine particular thresholds of weather it did not identify any association with wind or low temperature weather warnings and higher rates of hip fracture in the Irish population. Examination of these warning types presented difficulties as multiple weather warnings for different types of weather overlapped on a number of days. It is difficult to accurately compare weather events and parameters across countries and continents, which may contribute to the disagreement in literature with studies citing snow and low temperatures as a risk factor tending to have low temperatures and higher snowfall such as Canada, while Ireland has a more temperate climate and heavy snow fall would be infrequent.

## Strengths and limitations

This study utilised two national databases to investigate the relationship between hip fractures and weather warnings in the Republic of Ireland. This enabled capture of all IHFD recorded hip fractures occurring in association with weather warnings and comparison to rates in their absence. This, to the authors' best knowledge, is the first study using national data for both weather warnings and hip fractures to investigate a relationship between the two. While this study allowed assessment of greater data than many previous studies, an accurate assessment of relationships was limited by the small number of red warnings and the smaller population of Ireland with only 7 years of data available.

It also must be acknowledged that there were some weaknesses to the study. The location of each hip fracture geographically does not form part of the IHFD data and so an assumption was made that the fracture occurred in the individual’s county of residence. With frequent travel between counties for leisure or healthcare purposes this may not be accurate in all cases, however as the majority of hip fractures occur at home this should provide good accuracy. In the case of individuals registered as residing outside of Ireland we assumed the fracture to have occurred within the county of the hospital they attended, which may not be accurate with many orthopaedic units serving a catchment area spanning multiple counties. The mechanism of injury is also not recorded, however the majority of these injuries happen in the home [32].

In Ireland, there is no retrospective verification of weather warnings and whether predicted conditions occurred. This is due to the fact that warnings are not made purely based on specific thresholds but also largely on the perceived risk to the population and potential impact. As a result it cannot be guaranteed that for each warning the specified weather severity reached a designated level, however our findings suggest an impact of a warning being in place. There is potential for type 1 error due to multiple testing across warning elements/severities.

The IHFD only collects hip fracture data on patients aged ≥ 60 years in public hospitals. As a result younger hip fractures, which frequently represent higher energy trauma will not be included. Ireland has separate public and private healthcare systems, however the IHFD only collates data from public hospitals. A small number of hip fractures may have been treated in private institutions; however, it is unlikely that weather warnings would have an impact on the proportion of fractures treated in private institutions.

## Conclusion

An increased incidence of hip fractures appears to occur during weather warnings, in particular yellow and orange weather warnings, while for the most severe red warnings, this increase may be deferred until the days after the warning. This has implication for planning of emergency department staffing, and consideration should be given when preparing for periods of extreme weather, ensuring sufficiently resources. 

### Supplementary Information

Below is the link to the electronic supplementary material.

Supplementary file1 (DOCX 31 KB)

## Data Availability

The weather warning data that support the findings of this study are available MET Éireann and www.met.ie. Restrictions apply to the availability of the hip fracture data used for this study which were supplied by the National Office of Clinical Audit.

## References

[1] Wilers C, Norton N, Harvey NC (2022). Osteoporosis in Europe: a compendium of country-specific reports. Arch Osteoporos.

[2] National Office of Clinical Audit (2021) Irish hip fracture database national report 2020. Exectuvie Summary p 8

[3] Kanis JA, Norton N, Harvey NC (2021). SCOPE 2021: a new scorecard for osteoporosis in Europe. Arch Osteoporos.

[4] National Office of Clinical Audit (2021) Irish hip fracture database national report 2020. Chapter 7: Outcomes p 90

[5] Williamson S, Landeiro F, McConnell T, Fulford-Smith L, Javaid MK, Judge A (2017). Costs of fragility hip fractures globally: a systematic review and meta-regression analysis. Osteoporos Int.

[6] Menzin J, Wygant G, Hauch O, Jackel J, Friedman M (2008). One-year costs of ischameic heart diseaase among patients with acute coronary syndrome: findings from a multi-employer claims database. Curr Med Res Opin.

[7] Mercaldi CJ, Siu K, Sander SD, Walker DR, Wu Y, Li Q, Wu N (2012) Long-term costs of ischemic stroke and major bleeding events among medicare patients with nonvalvular atrial fibrillation. Cardiol Res Pract 2012:645469. 10.1155/2012/645469 10.1155/2012/645469 PMC3467774 23082276

[8] Pinkerton KE, Felt E, Riden HE (2019). Editorial: Extreme Weather resulting from global warming is an emerging threat to farmworker health and safety. J Agric Saf Health..

[9] Singh MS, Kuang Z, Maloney ED, Hannah WM, Wolding BO (2017). Increasing potential for intense tropical and subtropical thunderstorms under global warming. Proc Natl Acad Sci USA.

[10] Mirsaeidi M, Motahari H, Khamesi MT, Sharifi A, Campos M, Schraufnagel DE (2016). Climate change and respiratory infections. Ann Am Thorac Soc.

[11] Rossati A (2017). Global Warming and its health impact. Int J Occup Enviro Med.

[12] MacKenzie JS, Banskota B, Sirisreetreerux N, Shafiq B, Hasenboehler EA (2017). A review of the epidemiiology and treatment of orthopaedic injuries after earthquakes in developed countries. World J Emerg Surg.

[13] Franklin Sechriest V, Lhowe DW (2008). Orthoapedic care aboard the USNS Mercy during Operation Unified Assistance after the 2004 Asian tsunami. A case series. J Bone Jt Surg Am.

[14] Lanham N, Cockelman K, Lopez F, Serra MM, Scanlan B (2020). Orthoapedic care provided by the 14th combat support hospital in support of humanitarian and disaster relief after hurricane Maria in Puerto Rico. World J Orthop.

[15] Nau BJ, Woolley PM, Vertilus R (2018). Orthopaedics in Haiti. J Bone Jt Surg Am.

[16] Bortolin M, Morelli I, Voskanyan A, Joyce NR, Ciottone GR (2017). Earthquake-related orthopaedic injuries in adult population: a systematic review. Prehosp Disaster Med..

[17] Mazzucchelli R, Crespí-Villarías N, Pérez-Fernández E, Durbán Reguera ML, Guzón Illescas O, Quirós J, García-Vadillo A, Carmona L, Rodriguez-Caravaca G, Gil de Miguel A (2018) Weather conditions and their effect on seasonality of incident osteoporotic hip fracture. Arch Osteoporos 13(1):28. 10.1007/s11657-018-0438-4 10.1007/s11657-018-0438-4 29546463

[18] Hayashi S, Noda T, Kubo S (2019). Variation in fracture risk by season and weather: a comprehensive analysis across age and fracture site using a National Databse of Health Insurance Claims in Japan. Bone.

[19] Tenías JM, Estarlich M, Crespo E, Román-Ortiz C, Arias-Arias A, Ballester F (2015) Short-term relationship between hip fracture and weather conditions in two Spanish health areas with different climates. J Environ Public Health 2015:395262. 10.1155/2015/395262 10.1155/2015/395262 PMC4338400 25759722

[20] Masterson E, Borton D, Obrien T (1993). Victims of our climate. Injury.

[21] MET Éireann. Weather warnings explained. Available from https://www.met.ie/weather-warnings. Accessed 10 Nov 2021

[22] MET Éireann (2021) Ireland’s open data portal- archived weather warning datasets. Available from https://data.gov.ie/dataset/archived-weather-warnings. Accessed 17 Mar 2021

[23] Turner RM, Hayden A, Dunsmuir WTM, French CF (2011). Air Temperature and the incidence of fall-related hip fracture hospitalisation in older people. Osteoporos Int.

[24] Levy AR, Bensimon DR, Mayo NE, Leighton HG (1998). Inclement weather and the risk of hip fracture. Epidemiology.

[25] Lau EM, Gillespie BG (1995). The seasonality of hip fracture and its relationship with weather conditions in New South Wales. Aust J Public Health.

[26] Emaus N, Olsen LR (2011). Hip Fractures in a city in Northern Norway over 15 years: time trends, seasonal variation and mortality: the Harstad Injury Prevention Study. Osteoporos Int..

[27] Román-Oritz C, Tenías JM, Estarlich M, Ballester F (2015). Systematic review of the association between climate and hip fractures. Int J Biometeorol.

[28] Central Statistics Office (2017) Census 2016 profile 3- an age profile of Ireland. Available from https://www.cso.ie/en/csolatestnews/presspages/2017/census2016profile3-anageprofileofireland/. Accessed 30 Mar 2021

[29] Chevalley T, Hermann FR, Delmi M (2002). Evaluation of the age-adjusted incidence of hip fractures between urban and rural areas: the difference is not related to the prevalence of institutions for the elderly. Osteoporos Int.

[30] Mannius S, Mellström D, Odén A (1987). Incidence of hip fracture in western Sweden 1974–1982 Comparison of rural and urban populations. Acta Orthop Scand.

[31] Nursing Homes Ireland. Private and voluntary nursing home survery results 2019/2020. Chapter 5.2- Supply Growth p 17

[32] National Office of Clinical (2020) Major trauma audit: national report 2018. Dublin: National Office of Clinical Audit; 2020

[33] Tenías JM, Estarlich M, Fuentes-Leonarte V et al (2009) Short-term relationship between meteorological variables and hip fractures: an analysis carried out in a health area of the Autonomous Region of Valencia, Spain (1996–2005). Bone 45(4):794–8.10.1016/j.bone.2009.06.022 10.1016/j.bone.2009.06.022 19563926

