# Supplementary material for: The association between weather warnings and hip fractures in the Republic of Ireland
Source: Arch Osteoporos. 2023 Apr 21;18(1):53. doi: 10.1007/s11657-023-01243-9 (PMC10121515; doi:10.1007/s11657-023-01243-9)
Supplement: Supplementary file 1 — Supplementary file1 (DOCX 31 KB) [file 11657_2023_1243_MOESM1_ESM.docx]

**Supplementary materials**

Supplementary table 1 Sensitivity analysis for panel data analysis and case crossover analysis considering warnings to affect only the same days they are in place, or with a lag of one day

|  | **Same day only** | | **Lag of 1 day** | |
| --- | --- | --- | --- | --- |
|  | **Adjusted incidence rate ratio*** | **P value** | **Adjusted incidence rate ratio*** | **P value** |
| **Panel data analysis** |  |  |  |  |
| Red | 0.92 (0.70 to 1.22) | 0.569 | 1.14 (0.88 to 1.46) | 0.313 |
| Orange | 1.24 (1.10 to 1.41) | 0.001 | 1.18 (1.03 to 1.34) | 0.013 |
| Yellow | 1.09 (0.99 to 1.21) | 0.082 | 1.06 (0.96 to 1.17) | 0.241 |
|  | **Adjusted odds ratio**** | **P value** | **Adjusted odds ratio**** | **P value** |
| **Case crossover analysis** |  |  |  |  |
| Red | 0.90 (0.66 to 1.21) | 0.484 | 1.12 (0.84 to 1.48) | 0.437 |
| Orange | 1.29 (1.12 to 1.49) | 0.001 | 1.24 (1.07 to 1.43) | 0.004 |
| Yellow | 1.15 (1.03 to 1.29) | 0.012 | 1.12 (1.00 to 1.25) | 0.05 |

* Poisson regression with county population aged 50 years and over as exposure, adjusted for season, year, and warning element.

** Conditional logistic regression matching case and control days for each individual, adjusted for warning element.

Supplementary table 2 Summary of hip fracture events and unadjusted incidence rates for days affected by weather warnings by warning element.

|  | Events | Exposure (million person days) | Incidence rate | Incidence rate difference (95% CI) | Incidence rate ratio (95% CI) | p value |
| --- | --- | --- | --- | --- | --- | --- |
| **No warning** | 15850 | 2688.8 | 5.89 | - | - | - |
| **Wind** | 2481 | 416.2 | 5.96 | 0.07  (-0.19 to 0.32) | 1.01  (0.97 to 1.06) | 0.601 |
| **Rainfall** | 1982 | 345.1 | 5.74 | -0.15  (-0.42 to 0.12) | 0.97  (0.93 to 1.02) | 0.271 |
| **Snow-Ice** | 1207 | 186.2 | 6.48 | 0.59  (0.21 to 0.96) | 1.10  (1.04 to 1.17) | 0.002 |
| **Low Temperature** | 678 | 106.6 | 6.36 | 0.46  (-0.02 to 0.95) | 1.08  (1.00 to 1.16) | 0.055 |
| **Fog** | 486 | 79.3 | 6.13 | 0.24  (-0.32 to 0.79) | 1.04  (0.95 to 1.14) | 0.123 |
| **Thunder** | 250 | 40.2 | 6.22 | 0.33  (-0.45 to 1.10) | 1.06  (0.93 to 1.20) | 0.396 |
| **High Temperature** | 193 | 38.8 | 4.97 | -0.92  (-1.63 to -0.22) | 0.84  (0.73 to 0.97) | 0.016 |
| **Flooding** | 18 | 2.9 | 6.22 | 0.33  (-2.55 to 3.20) | 1.06  (0.63 to 1.67) | 0.791 |
